# Foundation dentists’ attitudes and experiences in providing dental care for dependant older adults resident in care home settings

**DOI:** 10.1038/s41405-024-00285-6

**Published:** 2025-01-08

**Authors:** H. Raison, H. Parsley, Z. Shah, T. D. Manangazira, Y. Dailey

**Affiliations:** 1 Academic Clinical Lecturer in Dental Public Health, Institute of Population Health, The University of Liverpool, Liverpool, UK; 2 Healthcare Public Health Senior Project Manager, Public Health Directorate, NHS England North West, Manchester, UK; 3 Foundation Dentist NW, Public Health Directorate, NHS England North West, Manchester, UK; 4 Lead Consultant Dental Public Health, Public Health Directorate, NHS England North West, Manchester, UK

**Keywords:** Gerodontics, Preventive dentistry

## Abstract

**Introduction:**

There is a continued increase of older dependant adults in England. Foundation Dentists (FDs) are often the dental workforce being tasked with providing dental care to dependant older adults resident in care home settings. This study explores whether FDs have the experience and confidence to deliver this.

**Aim:**

This service evaluation aimed to explore FDs’ attitudes, perceptions and experiences delivering dentistry to dependant older adults’ resident in care home settings; to help inform workforce and service delivery planning.

**Methods:**

All North West England (NW) FDs were invited to complete a semi-structured questionnaire at a regional study session. Results were analysed using descriptive and thematic analysis.

**Results:**

There were 93 (80.1%) respondents, with the majority aged 20–24 years old (56, 60.2%), female (57, 61.3%) and with an United Kingdom undergraduate dental degree (88, 94.6%). Most respondents had no experience in delivering care in a care home setting at either undergraduate (85, 91.4%) or FD level (84, 90.3%). Only 14 respondents (15.1%) reported confidence to deliver dentistry in a care home setting.

**Conclusion:**

To deliver dental care for dependant older adults resident in care home settings, FDs require additional training and clinical support. There is a need to review the undergraduate dental curriculum and NHS postgraduate training programmes to increase knowledge and skills for this vulnerable group.

## Introduction

The English population profile is changing considerably, with a significant aging population. For example, the percentage of UK adults aged 65 years and over is projected to increase from 18% of the total population in 2016 to 26% in 2066 [1]. Furthermore, it is projected that the number of older people in England with dementia will increase 108% by 2040, contributing to a 166% increase in care home residents [2].

Dependant older adults residing in care home settings are a particular vulnerable group within the population. They rely on others for personal care and access to dental services and often present with complex oral health needs that require comprehensive and holistic care [3–7]. For example, comorbidities often exacerbate the oral health needs of the older population with conditions such as diabetes, hypertension, rheumatoid arthritis, Alzheimer’s and Parkinson’s having the potential to impact negatively upon an individual’s oral health [4]. This makes this subgroup of the population at higher risk for oral disease.

National clinical guidelines have been developed, which outline the requirements across the health and social care system, for managing and supporting the oral health for older patients. These include guidelines set out by the National Institute for Health and Care Excellence (NICE NG 48) and the NICE quality standard 151 (QS151) [8, 9]. To deliver care in line with clinical guidelines, a suitable dental workforce is required. With increasing pressures within the NHS England primary dental care workforce, Foundation Dentists (FDs) are often identified as suitable dental professionals to deliver such care (i.e. confident to deliver Tier 1 dental care to dependant older adults within care home settings).

FDs are qualified dentists in the first year(s) of postgraduate dental training. Dental Foundation Training is mandatory for joining the NHS performer list to provide NHS dental care. The training scheme has the purpose of enhancing clinical and administrative competence, as well as promoting high standards through relevant postgraduate training. They are remunerated through a set salary and represent a stable workforce (i.e. there are a constant number of FDs allocated across England each academic year). FDs are often seen as key players for the delivery of dental programmes for dependant older adult resident in care home settings, but exploration of their training and confidence to complete such initiatives, to the authors’ understanding, has never been previously explored.

## Aim

This service evaluation aimed to explore the attitudes and experiences of FDs delivering dentistry to dependant older adults resident in care home settings; to help inform workforce and service delivery planning.

## Objectives

- To investigate the experiences of FDs providing dental care for dependant older adults resident in care homes in both the dental and care home settings.
- To explore the extent to which undergraduate and foundation training has prepared (in terms of knowledge and confidence) FDs for providing care for dependant older adults in dental practice and care home settings.
- To investigate the training needs of FDs in providing dental care for dependant older adults resident in care home settings, including specific topics of interest and preferred modes of delivery.

## Methods

This service evaluation recruited FDs currently enrolled in the Dental Foundation Training Programme in the North West of England (NW) in 2023-2024 (excluding the 2 NW FDs on placement within the regional Dental Public Health Team). FDs were invited to participate in the service evaluation and complete a semi-structured questionnaire at their regional study day, with follow-up invite sent via email.

Participants self-completed a baseline, online questionnaire using Microsoft Forms (an online management tool) (Appendix 1). Adopting a mixed-methods approach, questions explored the FDs’ demographic characteristics and their professional attitudes and experiences of dental care provision for dependant older adults residing in care homes. The questionnaire was designed via a working group which included 2 NW FDs on placement with the regional Dental Public Health Team, an NIHR Academic Clinical Lecturer and a consultant in Dental Public Health, as well as a senior programme manager (with outreach dental student teaching experience) within NHS England NW Public Health directorate. All responses were anonymised (with FDs identified with codes P1-P93). The questionnaire took 10 min to complete. To ensure validity and reliability, the questionnaire was piloted with two FDs outside the NW, with amendments made as appropriate (e.g. amendments made to three questions; two required clarification and one question was removed) [10].

Data analysis was completed using descriptive analysis with all categorical variables presented as numbers and percentages. Open-ended questions were analysed using thematic analysis (i.e. within the FDs’ responses, identifying, analysing and interpreting emerging patterns) [11]. Using an inductive approach, descriptive coding was used to identify themes from the responses. Constant reflection of the service evaluation team’s positionality was required, as some members were FDs. A sample of the data was double-coded to ensure validity [12]. Themes were refined and named to give the overarching key findings. Member checking (i.e. interpretations and findings from the open-ended questions were shared back to participants for confirmation to ensure true representation) was completed to ensure accuracy and validity [13].

## Results

Of the 116 NW FDs, 93 (80.1%) responded to the semi-structured questionnaire at their regional study day, with the majority being aged 20-24 years old (56, 60.2%) and female (57, 61.3%). Almost all, 88 (94.6%) participants, completed their undergraduate dental degree within an United Kingdom university.

During their undergraduate training, 85 (91.4%) participants never had the opportunity to visit a care home. Within FD training, again, experience was very limited. Many (84, 90.3%) had never delivered care in a care home setting but did have the opportunity to deliver care to a care home resident within their dental practice (44, 47.3%), with 35 participants (37.6%) providing active dental treatment. Confidence to provide dental care for dependant older adults was higher when conducted within a dental practice setting (61, 65.6%) compared to the care home setting (14, 15.1%).

**Fig. 1:**
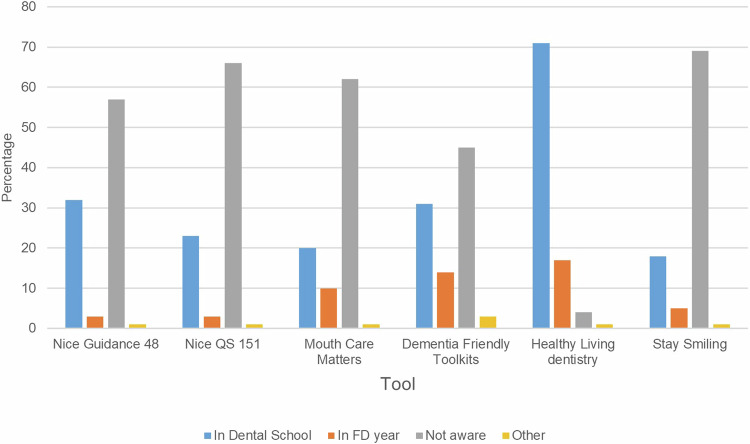
Foundation Dentist (FD) knowledge of guidance / tools relating to dependant older adults residing in care home settings.

Knowledge around tools relating to dependant older adults, such as NICE Guidance 48, NICE quality standard 151 and Mouth Care Matters, was also limited within this cohort of FDs (Fig. 1). For example, 57 participants (61.3%) were not aware of NICE Guidance 48 and 62 participants (66.7%) were unaware of the national Mouth Care Matters programme. Predominantly, observations (74, 79.5%) and workshops (75, 80.6%) were the training methods of choice to upskill and develop confidence within the FDs to deliver dental care for dependant older adults in the care home setting.

FDs’ views on the value and relevance of dentistry for the dependant older population on their practice and career development was overall positive. The majority (69, 74.2%*)* agreed that dentistry for the dependant older population residing in care homes was relevant and applicable to their current and future practice but 70 (75.3%) reported that it requires specific knowledge and skills that were different from general dentistry. Furthermore, 65 (69.9%) reported that dentistry for the dependant older adult was challenging and stressful; whilst 54 (58.1%) stating that it was rewarding and enjoyable.

Two main themes emerged from the qualitative data regarding the challenges and barriers to providing dental care for dependant older adults resident in care home settings within a dental practice: “complex, medical patient” and “limitations of practice facilities”. In contrast, “lack of clinician experience” was the key theme regarding the challenges and barriers to dental care provision for dependant older adults within the care home setting. Each of these themes will be described in turn, with example quotations included in italics for each.

### Challenges and barriers within dental practice

- Complex, medical patient
  Participants discussed the medical complexities of these patients and how this has a negative impact upon dental care provision. For example, participants focused on patients’ inability to lie back on the dental chair for extended periods of time, polypharmacy implications including dry mouth, and issues with verbal and written communication. However, the main impact highlighted was consent. FDs were repeatedly unsure about how valid consent is obtained or who gives valid consent for this cohort of patients.
  *“*…*I also believe consent is a barrier as sometimes it is not clear who the POA [Power of Attorney] is and so non-urgent tx must be delayed till after best interest meetings are done*.*”* – P49
- Limitations of dental practice facilities
  Issues relating to dental practice access were also highlighted. Stairs were a key concern, with participants reporting that this would hinder the care home residents’ ability to access certain dental treatment rooms within the dental practice. Limitations of some dental practices to accommodate wheelchairs in certain clinics and have suitable space for carers and family members were also highlighted.
  *“My surgery is upstairs, and the stairs are very steep, so no frail elderly patients get booked with me”* – P24

### Challenges and barriers within care homes

- Lack of clinician experience
  Lack of experience of delivering dental care and/or prevention within the care home setting throughout undergraduate and FD training was a strong theme which emerged. Participants reported how gaining this experience within the FD year “*wasn’t possible”* as this wasn’t something their dental foundation practices offered. This lack of experience within the care home also raised questions around the range of dental care which could be provided, and how, within the care home setting.
  “*Never had any experience so unsure how it would work – I assume a lot would be preventive tx as fillings etc would be difficult to do without a dental surgery” –* P31

## Discussion

With a rapidly growing aging population, it is important to consider how dental needs can be met for dependant older adults residing in care home settings [14]. There is often the suggestion when considering oral health improvement and dental access initiatives, both nationally, regionally and within Integrated Care Boards (ICBs), that FDs represent an appropriate workforce to deliver such care because the workforce numbers are stable each year to facilitate planning; and they are salaried from a training rather than a dental commissioning budget. However, this service evaluation demonstrates some important findings which may impact upon the suitability of FDs to deliver dental care for dependant older adults who are resident in care home settings.

### Experiences, knowledge and confidence of FDs to provide dental care for the dependant older adult residing in care home settings

During undergraduate training, there is limited experience in treating dependant older adults who are resident in care homes either within the dental school or the care home setting. This does improve slightly during the foundation year, with more participants treating dependant older adults who reside in care homes, but this experience is mostly limited to provision within the dental practice setting. This lack of contextually rich concrete experience limits FDs’ learning in relation to this vulnerable group [15]. Using the Kolb’s learning cycle, the lack of “active experimentation”, offered through treating residents from care homes throughout undergraduate and FD training, hinders the translation of theoretical knowledge to the applied setting [16, 17]. This has perhaps been most evident when considering the issue of valid consent. Whilst students understand the theory in relation to consent to dental treatment, one of the barriers to the provision of dental treatment within the dental practice context was *how (*in the practical sense) valid consent can be achieved for this cohort (particularly those patients with neurodegeneration).

The lack of “concrete experience” may have also contributed to the low confidence reported in providing dental care for dependant older adults residing in care home settings [18]. Increasing confidence, through experiential learning (e.g. simulations relating to specific real-world clinical problems, case-based discussions) has the potential to change FDs’ perceptions of training around dental care delivery for dependant older adults living in care homes [19]. A strong preference for training methods such as observations and workshops were observed in this study, fitting into the experiential method of learning. Furthermore, increased confidence improves clinical competence to deliver appropriate dental care [20]. Whilst clinical dental experience and high confidence have no predictive value in quality of dental care delivered, there is a need to consider how, at both undergraduate and FD training levels, experience and confidence for treating dependant older adults who reside in care home settings could be improved [21]. Experiential learning should be supplemented with specific knowledge around national and regional evidence-based guidance for dependant older adults as current levels of awareness within FDs are low [5, 8]. This will help to facilitate the delivery of evidence-based care across the primary dental care workforce.

### FDs’ views on the value and relevance of providing dental care for the dependant older adults residing in care homes on their practice and career development

Overall, FDs’ views on the value and relevance of dentistry for dependant older adults residing in care homes was positive. Many agreed that it was relevant and applicable to their current and future practice, and an important and growing field of dentistry, which offered opportunities for professional growth and development. Many felt that dentistry for the dependant older population residing in care home settings “required specific knowledge and skills that are different from general dentistry”. Furthermore, dental care provision was deemed challenging and stressful but also rewarding and enjoyable. The national English guide for the commissioning of Special Care Dentistry outlines differing care descriptors based on which clinician should provide the clinical treatment [22]. Level 1 care outlines what should be delivered by the completion of undergraduate and FD training. This helps to determine an objective measure of care providers and should help to address FD concerns about the need for specialist treatment.

Limitations to this service evaluation were noted. Only NW FDs were invited to respond to the questionnaire during their regional study day, due to the limitations of this being a service evaluation and not a research project. There may be potential for selection bias among those who responded to the questionnaire, as non-respondents might have had less experience in treating this patient group or may not have considered it relevant or important.

Further work is therefore needed to explore how the findings and implications of this service evaluation can be applied across the English FD workforce. This is particularly important when considering regional variations in dental care delivery for dependent older adults living in care home settings which may impact on FDs’ experience and confidence in treating this patient group. Adopting this approach could facilitate shared learning at a national level, allowing best practices from one region to be disseminated. However, given the high response rate, with FD trainees wanting to report on their training experience and how this could positively influence further training / experiences, and the representation of the sample regarding training from different UK dental schools, gender and age, the insights gathered from this service evaluation would be anticipated to be reflective of the current FD workforce.

The findings of this study raise questions relating to the experience of the current, younger primary dental care workforce, who may in the future become FD trainers, yet they themselves may not have received training or experience for providing dentistry to this cohort. This is particularly important over the last 5 years, where the waves of the COVID-19 pandemic have prevented access to care homes and/or residents for preventive / active dental treatment. Furthermore, it might be pertinent to undertake a broader assessment of primary care dental workforce training needs in relation to providing dental care for dependant older adults residing in care home settings, given the significant external and contextual changes over recent decades including changes in oral health profiles, life expectancies etc.

Finally, limitations on cross-sectional questionnaire should be noted. It captures data at a single point in time, which does not demonstrate changes or trends in experience and/or confidence over time. Additionally, it may be subject to response bias, as it relies on self-reported data.

## Conclusion

Whilst FDs represent a stable, consistent dental workforce for the delivery of oral health improvement and dental access initiatives for older dependant adults residing in care home settings, they have reportedly limited experience and confidence to deliver such care, especially within the care home setting. Additional training and/or clinical support would be required to enable these clinicians to deliver appropriate dental care for this population cohort. The application of the findings of this service evaluation will vary across deaneries and localities; however, as an overall recommendation, we advocate incorporating experiential learning by a skilled and confident dental workforce, which could include a visit to a care home to gain a better understanding of the setting, its operations, and challenges. Furthermore, there is a call to consider reviewing the undergraduate dental curriculum and NHS postgraduate training programmes, across the United Kingdom, to increase both knowledge and clinical skills relating to treating dependant older adult residing in care home settings.

## Supplementary information

Appendix 1

## Data Availability

The data that support the findings of this study are available from the corresponding author, HR, upon reasonable request.
