# Supplementary material for: Foundation dentists’ attitudes and experiences in providing dental care for dependant older adults resident in care home settings
Source: BDJ Open. 2025 Jan 8;11:3. doi: 10.1038/s41405-024-00285-6 (PMC11711489; doi:10.1038/s41405-024-00285-6)
Supplement: Supplementary file 1 — Appendix 1 [file 41405_2024_285_MOESM1_ESM.pdf]

Appendix 1. Self-completed online questionnaire

Question 1. What is your age?

- 20-24 years
- 25-30 years
- 30+ years

Question2. How would you describe your gender?

- Female
- Male
- Prefer not to say
- Other

Question 3. Where did you complete your dental degree?

- UK
- Other

Question 4. Aside from undergraduate and foundation training, do you have other health and/or social care experience?

- Yes
- No

Question 5. If you answered yes in the previous question, can you give more details?

Question 6. Did you have the opportunity to visit a care home during your undergraduate training? (you can select more than one answer)

- Never
- Observed
- Conducted an oral health assessment
- Delivered preventive care
- Delivered treatment

Question 7. Have you had the opportunity to deliver care in a care home setting during FD training? (you can select more than one answer)

- Never
- Observed
- Conducted an oral health assessment
- Delivered preventive care
- Delivered treatment

Question 8. Have you had the opportunity to deliver care in a practice setting to a patient from a care home during FD training (you can select move than one answer)

- Never
- Observed
- Conducted an oral health assessment
- Delivered preventive care
- Delivered treatment

Question 9. Are you aware of any of the following tools (please reply for each tool)?

If you are aware, please select how you were made aware (options given: not aware; in dental school; in FD year; Other

- NICE guidance48 (NG48)
- NICE quality standard 151 (QS151)
- Mouth care matters
- Dementia friendly toolkits such as Cgdent, BDA
- Healthy Living Dentistry
- Stay Smiling
- Other

Question 10. If you selected ‘other’ in the question above, please give further details below

Question 11. How confident do you feel in providing dental care for dependant older adults in: (answers range from not confident; confident; very confident)

- A care home setting
- Practice setting

Question 12. What do you consider are the main current challenges or barriers that you face in providing dental care for dependant older patients in practice?

Question 13. What do you consider are the main current challenges or barriers that you face in providing dental care for dependant older patients in care home setting?

Question 14. What methods of further training on dentistry for the dependent older adult population in a care home setting you like to have in the future? Please select all that apply: (Not interest, interested, very interested)

- Online courses
- Webinars
- Workshops
- Conferences
- Mentorship
- Observation
- Speciality training programme
- Research

Question 15. In your opinion, what topic(s) would you like further training in?

Question 16. To what extent do you agree or disagree with the following statements on dentistry for the dependant older adult and its value and relevance for your practice and career development? Please rate your level of disagreement (1) or agreement (5) on the scale

- Relevant and applicable to my current and future practice
- Important and growing field of dentistry
- Rewarding and enjoyable
- Requires specific knowledge and skills that are different from general dentistry
- Offers opportunities for professional growth and development
- Adequately recognised and valued by the dental profession and society
- Challenging and stressful

Question 17. Is there anything additional you would wish to share regarding managing a patient in a care home setting?
